# A Retrospective Study Analysing the Outcomes of Anxiety, Depression, and Sleep Disorders in Post-Chemotherapy Patients With Breast Cancer Based on Symptom-Targeted Supportive Psychological Nursing

**DOI:** 10.62641/aep.2270

**Published:** 2026-10-15

**Authors:** Yanyan Shi, Yu Zhou, Jin Yan, Ruipeng Zhao, Qing Zhang, Jing Xu

**Affiliations:** ^1^Department of Thyroid and Breast Surgery, The Affiliated Huaian No.1 People's Hospital of Nanjing Medical University, 223300 Huaian, Jiangsu, China; ^2^Department of Nursing, The Affiliated Huaian No.1 People's Hospital of Nanjing Medical University, 223300 Huaian, Jiangsu, China

**Keywords:** breast cancer, post-chemotherapy, supportive psychological nursing, depression, anxiety

## Abstract

**Background:**

To analyse the differences in supportive psychological nursing care received by patients with breast cancer during and after chemotherapy.

**Methods:**

A retrospective cohort study was conducted, selecting 152 patients with breast cancer who received standardised chemotherapy and completed follow-up at our hospital between April 2022 and April 2025. Patients were divided into a supportive care group (n = 76) and a routine care group (n = 76) based on differences in supportive psychological nursing care in their medical records and nursing documentation. The levels of depression, anxiety, psychological distress and sleep quality after chemotherapy were then compared between the two groups.

**Results:**

Depression and anxiety levels in the supportive care group were lower than those in the routine care group (*p* < 0.05). The psychological distress thermometer score and insomnia severity index scores in the supportive care group were significantly lower than those in the routine care group (*p* < 0.05). Analysis of disease coping strategies showed that patients in the supportive care group scored significantly higher on the coping dimension than those in the routine care group and lower on the avoidance and yielding dimensions (*p* < 0.05).

**Conclusions:**

Supportive psychological nursing care was associated with improved depression and anxiety in patients with breast cancer after chemotherapy.

## Introduction

Breast cancer is one of the most common cancers among women worldwide. Thanks to the widespread adoption of early screening methods and the continuous improvements in comprehensive treatment strategies, the overall survival rate has substantially increased [1]. Chemotherapy is an important part of the comprehensive treatment system for breast cancer and is invaluable in reducing the risk of recurrence and improving prognosis. However, its accompanying adverse reactions affecting multiple systems and psychosocial impacts are becoming increasingly prominent [2, 3]. Previous clinical practice and epidemiological data show that patients with breast cancer experience substantially higher rates of negative emotions, such as depression and anxiety, after chemotherapy than the general population. Such emotional disorders directly affect the quality of life of patients and may indirectly interfere with disease recovery by disrupting sleep structure, immune function and neuroendocrine homeostasis. In addition, persistent anxiety and depression are closely related to adverse outcomes such as decreased treatment compliance, interrupted follow-up and reduced efficiency of medical resource utilisation [4, 5, 6]. Therefore, the mental health status of patients with breast cancer after chemotherapy has become a key focus in tumor nursing and supportive treatment [7]. In clinical nursing practice, supportive psychological nursing is considered to have potential value in reducing patients’ psychological burden and promoting functional recovery. This nursing model covers multiple dimensions such as symptom management, psychological support, health education and social support coordination [8, 9, 10]. Currently, most studies focus on the design of prospective nursing plans or the evaluation of the treatment effect of single psychological indicators. However, there is still a lack of integrated research results based on the same cohort at the level of multidimensional psychological outcomes [11]. This study adopted a retrospective study design, using the hospital information system and nursing documents to analyse patients with breast cancer receiving chemotherapy between April 2022 and April 2025. The study compared the changes in patients’ depression and anxiety symptoms, as well as related psychosocial indicators, under different characteristics of supportive psychological nursing implementation. This study aims to explore the relationship between differences in the implementation of supportive psychological nursing and psychological outcomes. It also aims to provide an evidence-based foundation for the subsequent improvement and optimisation of nursing quality and psychological support strategies. Unlike previous studies that focused on single psychological indicators or prospective nursing plan designs, this study retrospectively analyses multidimensional psychological outcomes (depression, anxiety, sleep disorders, psychological distress and coping strategies) within the same cohort. This provides integrated evidence for supportive psychological nursing in post-chemotherapy patients with breast cancer.

## Materials and Methods

### General Information

A retrospective study was conducted involving patients with breast cancer who received standardised chemotherapy and completed follow-up at the Department of Oncology at the Affiliated Huaian No.1 People’s Hospital of Nanjing Medical University between April 2022 and April 2025. A total of 152 eligible patients with breast cancer were included in the study. Patients were divided into a supportive care group and a routine care group based on differences in supportive psychological nursing care in their medical records and nursing documentation, with 76 patients in each group. The study protocol was registered and the anonymity of all participants was guaranteed. All participants provided written informed consent, including prior general consent for the research-based use of their medical records during hospitalisation.

### Inclusion and Exclusion Criteria

#### Inclusion Criteria

(1) The patient was diagnosed with breast cancer by pathological examination; (2) The patient completed at least one full cycle of systemic chemotherapy within the study period; (3) The patient entered the follow-up stage after chemotherapy and completed the relevant psychological assessments (depression, anxiety, psychological distress, sleep disorders and coping strategies); (4) The patient had complete medical and nursing records and follow-up data; (5) The patient had basic communication skills and could complete the questionnaire independently or with guidance.

#### Exclusion Criteria

(1) The patient had a pre-existing diagnosis of severe depressive disorder, anxiety disorder or other serious mental illness (based on the International Classification of Diseases, 10th revision (ICD-10) or Diagnostic and Statistical Manual of Mental Disorders, 5th edition (DSM-5) criteria), diagnosed prior to the breast cancer diagnosis; (2) The patient had other malignant tumours or serious systemic diseases, such as heart, liver or kidney dysfunction.

### Study Design

#### Routine Care Group

Patients in the routine care group received routine nursing care from the oncology department of our hospital after chemotherapy [12]. (1) Nursing staff regularly monitored the patients’ vital signs according to routine nursing procedures. This included monitoring changes in body temperature, pulse, respiration and blood pressure. Staff also inquired about common symptoms of discomfort after chemotherapy, such as fatigue, nausea and loss of appetite. Any abnormalities were reported to the physician promptly. (2) Nursing staff assisted the patients in taking chemotherapy-related drugs as prescribed by the doctors, checking the drug name, dosage and administration time, and observing any adverse reactions after administration. (3) Before discharge, nursing staff provided patients and their families with routine oral education, including post-chemotherapy precautions, basic dietary principles, simple activity guidance and follow-up visit reminders. (4) On the day of discharge, the nursing staff assisted the patients in completing the discharge procedures and informed them of the follow-up time and related examination arrangements. The follow-up was mainly carried out by the patients themselves during follow-up visits to the hospital.

### Supportive Care Group

After chemotherapy, patients in the supportive care group received supportive psychological nursing support [13]. This approach was delivered by oncology nurses who had received standardised training in supportive psychological care (8 hours, including communication skills and strategies for addressing specific symptoms). Each in-hospital session lasted 15–25 minutes, and each telephone follow-up lasted 10–20 minutes. During hospitalisation, sessions were held once daily. After discharge, weekly telephone follow-ups were held for 4 weeks, with additional WeChat (Tencent Inc., Shenzhen, China) communication as needed. A structured protocol consisting of six core components (detailed below) was followed. Treatment fidelity was monitored through monthly supervision meetings and review of randomly selected nursing records. The specific components were as follows: (1) Targeted therapy for depressive symptoms: during hospitalisation and follow-up, nursing staff continuously monitored patients’ emotional state through face-to-face communication. During the nursing process, staff guided patients to express their true feelings about the disease, physical changes after chemotherapy and their outlook on the future through open-ended questions. They focused on whether patients exhibited symptoms such as low mood, loss of interest, self-blame or feelings of helplessness. During communication, the nursing staff helped patients process their emotional experiences by listening patiently, repeating the key points of the patient’s emotions, and confirming their feelings. They avoided simple comforting or denying the patient’s feelings. With the patient’s consent, the nursing staff explained the common occurrence of emotional fluctuations after chemotherapy, drawing on their previous experience. They guided patients to understand their own emotional reactions as normal psychological responses to the treatment, thereby reducing self-denial and isolation. For patients experiencing significant mood swings, nursing records indicate that the responsible nurse implemented targeted therapy for depressive symptoms through structured psychological communication during hospitalisation. (2) Targeted therapy for anxiety symptoms: in response to patients’ concerns about disease recurrence, persistent adverse reactions and uncertainty about future life after chemotherapy, nursing staff provided tiered health education during hospitalisation and discharge guidance. They explained the evolution of common physical reactions after chemotherapy, recovery time ranges and life factors that patients can manage themselves, tailoring their explanations to patients’ comprehension abilities and avoiding overly vague or fear-inducing expressions. Nursing records show that, during the educational process, nursing staff focused on helping patients distinguish between physical reactions that had occurred and concerns about what might happen in the future. This helped patients establish relatively clear recovery expectations and reduced anxiety experienced by misinformation by repeatedly confirming the patient’s level of understanding. Targeted therapy for anxiety symptoms was mainly implemented through face-to-face health education during hospitalisation and enhanced communication during the follow-up stage. Nursing records show that, during hospitalisation, health education communication related to systemic anxiety was conducted. During the follow-up period after discharge, anxiety scoring and explanatory guidance were carried out simultaneously with weekly follow-ups and the frequency of treatment was consistent with the frequency of follow-ups. (3) Targeted treatment for sleep disorders: in response to problems reported by some patients in their nursing records, such as difficulty falling asleep, frequent night-time awakenings, and decreased sleep quality, nursing staff inquired in detail about the patients’ sleep status, including sleep onset time, number of night-time awakenings, daytime rest, and bedtime habits. Based on this information, the nursing staff guided the patients to establish a relatively fixed schedule, reduce long-term bed rest during the day, and avoid prolonged use of electronic devices before bedtime. The nursing records also show that the nursing staff also guided patients to perform simple relaxation activities before bedtime, such as slow breathing exercises or listening to light music. They also made feasible suggestions to improve the sleep environment based on the patient’s living situation. During the follow-up stage, the nursing staff monitored changes in the patients’ sleep patterns and provided targeted reminders based on their feedback. For patients with sleep problems, the nursing staff analysed sleep status systematically during hospitalisation and provided personalised sleep behaviour guidance. During the follow-up stage after discharge, sleep changes were tracked through weekly follow-ups and reminders were provided. (4) Adjustment of disease coping strategies: During nursing communication and family support, nursing staff observed the patient’s attitude and behavioural responses to the disease. Nursing records show that when communicating with patients and their families, nurses emphasised the patient’s active role in the recovery process. They guided patients to focus on controllable behaviours in their daily life, such as managing their daily routine, regulating their emotions, and cooperating with follow-up care, rather than passively enduring the impact of the disease. Nurses avoided negative or threatening language, instead helping patients to develop a rational understanding of the disease process through factual explanations and practical guidance. At the same time, nurses explained to family members the importance of supportive companionship, encouraging them to focus on listening and companionship and to avoid blaming or avoiding the topic of the disease. (5) Continuous follow-up support: After discharge, the designated nurse will provide ongoing support based on the nursing records. Methods of follow-up include telephone contact, outpatient appointments and communication via WeChat. The first telephone follow-up takes place in the first week after discharge to assess the patient’s recent physical discomfort, emotional state and sleep status. A further follow-up takes place one month after discharge, focusing on changes in the patient’s psychological state and ability to adapt to daily life. Throughout the follow-up process, nurses will respond to any questions raised by the patient and provide relevant guidance and reminders based on changes in their psychological state. (6) Supportive psychological nursing care for the supportive care group began during the recovery period after chemotherapy had been completed and continued until the end of the follow-up phase after discharge. During hospitalisation, the responsible nurse conducted short-term psychological counselling sessions lasting about 15 to 25 minutes. After discharge, follow-up was conducted by telephone and supplementary communication was conducted via WeChat when necessary.

### Observation Indicators

#### Emotional State Analysis

The Patient Health Questionnaire Depression Scale was used. The Patient Health Questionnaire-9 (PHQ-9) [14] was used to analyse the level of depressive symptoms in patients. The PHQ-9 comprises nine items, each scored from 0 to 3, with a total score ranging from 0 to 27. It is primarily used to analyse the frequency of depressive symptoms experienced over the previous 2 weeks. 0 to 4 points indicates no depression, 5 to 9 points indicates mild depression, 10 to 14 points indicates moderate depression, and ≥15 points indicates moderate to severe depression. The total score range is 0 to 27 points. The higher the score, the more severe the depressive symptoms. This scale has good reliability and validity in cancer patients. The Cronbach’s α coefficient is approximately 0.86. The Generalised Anxiety Disorder 7-item scale (GAD-7) scale analyses the degree of anxiety symptoms in patients [15]. The scale comprises seven items, each scored from 0 to 3, with a total score range of 0 to 21. It is used to analyse the frequency and severity of anxiety symptoms experienced by patients over the past 2 weeks. The scoring criteria are as follows: 0 to 4 points indicates no obvious anxiety symptoms; 5 to 9 points indicates mild anxiety; 10 to 14 points indicates moderate anxiety; and ≥15 points indicates severe anxiety. The higher the score, the more severe the anxiety symptoms. The GAD-7 has good reliability and validity in patients with tumours and other chronic diseases. Its Cronbach’s α coefficients are typically reported to be between 0.88 and 0.92.

#### Level of Psychological Distress

The Distress Thermometer (DT) was used to measure psychological distress. The DT [16] analyses the overall level of psychological distress experienced by patients. The tool uses an intuitive numerical scoring method ranging from 0 to 10 points. 0 points means “no psychological distress” and 10 points means “extreme psychological distress”. The higher the score, the more severe the psychological distress.

#### Severity of Sleep Disorder

The Insomnia Severity Index (ISI) [17] was used to analyse the severity of patients’ sleep problems. The ISI comprises seven items, each scored from 0 to 4 points, giving a total score range of 0 to 28. It is mainly used to analyse difficulty falling asleep, sleep maintenance disorder and early awakening, as well as their impact on daytime function. The scoring criteria are as follows: 0 to 7 points indicates insomnia with no clinical significance; 8 to 14 points indicates mild insomnia; 15 to 21 points indicates moderate insomnia; and 22 to 28 points indicates severe insomnia. The higher the score, the more severe the insomnia. The Cronbach’s ( coefficient of the ISI for patients with tumours and chronic diseases is typically between 0.84 and 0.90, indicating good internal consistency.

#### Disease Coping Strategies

A Medical Coping Strategy Questionnaire (MCMQ) [18] analyses the psychological and behavioural patterns of patients coping with disease. The scale includes three dimensions: confrontation (score range 8–32), avoidance (7–28) and resignation (5–20). The higher the score in each dimension, the more evident the patient’s tendency towards that particular coping mode becomes. The Cronbach’s ( coefficients for the three dimensions are generally between 0.69 and 0.82, indicating an acceptable level of reliability.

### Statistical Analysis

Data analysis was performed using the statistical software SPSS 26.0 (IBM Corp., Armonk, NY, USA). The normality of continuous variables was tested using the Shapiro‒Wilk test. Normally distributed data are presented as mean ± standard deviation (mean ± SD). Independent-sample t-tests were used for between-group comparisons. Paired t-tests were used for within-group comparisons (pre-care vs post-care). Categorical data were compared using the χ^2^ test or Fisher’s exact test, as appropriate. For variables with more than two categories (e.g., tumour stage, educational level, marital status and physical activity level), the χ^2^ test was applied to the overall distribution across all categories. For repeated measures (PHQ-9, GAD-7, DT, ISI and MCMQ dimensions), paired t-tests were used for within-group comparisons and independent t-tests for between-group comparisons at each time point. No repeated-measure ANOVA was performed as only two time points were assessed. Exact *p*-values are reported rather than thresholds alone. Effect sizes (Cohen’s d for t-tests) are reported in the Results section. A two-tailed *p*-value of less than 0.05 was considered statistically significant.

Using G*Power 3.1 (Heinrich Heine University Düsseldorf, Düsseldorf, North Rhine-Westphalia, Germany) with α = 0.05, power = 0.80 and an assumed effect size d = 0.5 (moderate), a minimum of 64 patients per group was required. Considering a 10% dropout rate, 76 patients per group were enrolled.

## Results

### Comparison of General Information Between the Two Groups of Patients

Table 1 summarises the baseline demographic and clinical characteristics of the supportive care group (n = 76) and the routine care group (n = 76). The mean age was 51.32 ± 8.74 years in the supportive care group and 50.81 ± 9.14 years in the routine care group, with no statistically significant difference (t = 0.352, *p* = 0.72). Regarding tumour stage, the distribution of stages I, II and III was 18, 34 and 24, respectively, in the supportive care group, and 20, 32 and 24, respectively, in the routine care group (χ^2^ = 0.166, *p* = 0.92). The distribution of marital status distribution was comparable between the two groups (χ^2^ = 0.445, *p* = 0.80). In the supportive care group, 62 patients (81.6%) were married, while 14 patients (18.4%) were unmarried, divorced, or widowed. In the routine care group, 60 patients (78.9%) were married, while 16 patients (21.1%) were unmarried, divorced, or widowed. Similarly, educational level was distributed as follows: in the supportive care group, 21 patients had an education of up to junior high level, 29 had a high school education, and 26 had a college education or higher; in the routine care group, the corresponding numbers were 23, 27, and 26, respectively (χ^2^ = 0.162, *p* = 0.92). These results indicate that the two groups were well matched at baseline and that none of the differences reached statistical significance (all *p* > 0.05).

**Table 1.** **Baseline demographic characteristics of the two groups**.

Characteristic		Supportive care group (n = 76)	Routine care group (n = 76)	t/χ^2^	*p*
Age (years, mean ± SD)		51.32 ± 8.74	50.81 ± 9.14	0.352	0.72
Sex, n (%)				0.126	0.72
	Male	22 (28.9%)	24 (31.6%)		
	Female	54 (71.1%)	52 (68.4%)		
BMI (kg/m^2^, mean ± SD)		23.85 ± 3.21	24.12 ± 3.45	0.498	0.62
Tumour stage				0.166	0.92
	I	18 (23.7%)	20 (26.3%)		
	II	34 (44.7%)	32 (42.1%)		
	III	24 (31.6%)	24 (31.6%)		
Breast cancer subtype, n (%)				0.287	0.96
	Luminal A	28 (36.8%)	30 (39.5%)		
	Luminal B	26 (34.2%)	24 (31.6%)		
	HER2-enriched	12 (15.8%)	13 (17.1%)		
	Triple negative	10 (13.2%)	9 (11.8%)		
Marital status, n (%)				0.445	0.80
	Married	62 (81.6%)	60 (78.9%)		
	Unmarried/divorced/widowed	14 (18.4%)	16 (21.1%)		
Education				0.162	0.92
	≤Junior high	21 (27.6%)	23 (30.3%)		
	High school	29 (38.2%)	27 (35.5%)		
	≥College	26 (34.2%)	26 (34.2%)		
Smoking status, n (%)				0.107	0.74
	Current/former smoker	14 (18.4%)	16 (21.1%)		
	Non-smoker	62 (81.6%)	60 (78.9%)		
Alcohol consumption				0.276	0.60
	Current/former drinker	18 (23.7%)	21 (27.6%)		
	Never drinker	58 (76.3%)	55 (72.4%)		
Physical activity level, n (%)				0.284	0.87
	Low	32 (42.1%)	30 (39.5%)		
	Moderate	35 (46.1%)	38 (50.0%)		
	High	9 (11.8%)	8 (10.5%)		

BMI, body mass index; HER2, human epidermal growth factor receptor 2. There were no statistically significant differences between the two groups for any of the baseline characteristics (all *p* > 0.05).

### Comparison of Emotional States Between the Two Groups of Patients

The nursing process was divided into two stages: the in-hospital stage (from completion of chemotherapy to discharge) and the post-discharge follow-up stage (4 weeks after discharge). During the in-hospital stage, the pre-care assessment was conducted within 48 hours of completion of the last chemotherapy cycle. There were no significant differences in PHQ-9 and GAD-7 scores between the supportive care group and the routine care group (*p* > 0.05). The post-care assessment was conducted during the post-discharge follow-up stage, 4 weeks after discharge (corresponding to the completion of the 4-week telephone follow-up). The PHQ-9 and GAD-7 scores of patients in the supportive care group were lower than those in the routine care group at this stage, and the differences between the groups were statistically significant (*p* < 0.05). Intra-group comparisons showed that the PHQ-9 and GAD-7 scores of patients in the supportive care group decreased significantly between the pre-care and post-care assessments (*p* < 0.05). In contrast, no statistically significant difference was observed in the scores of patients in the routine care group between the two time points (*p* > 0.05) (Table 2).

**Table 2.** **Comparison of emotional state scores between the two groups of patients (mean ± SD, points)**.

Group	n	PHQ-9		GAD-7	
		Pre-care	Post-care	Pre-care	Post-care
Supportive care group	76	8.62 ± 2.71	6.21 ± 2.48^*^	7.93 ± 2.56	5.84 ± 2.17^*^
Routine care group	76	8.79 ± 2.84	8.73 ± 2.96	7.88 ± 2.61	7.69 ± 2.64
t		0.378	5.689	0.119	4.719
*p*		0.706	<0.001	0.905	<0.001
Cohen’s d			0.92		0.77

PHQ-9, Patient Health Questionnaire-9; GAD-7, Generalised Anxiety Disorder 7-item scale. Pre-care refers to the baseline assessment conducted within 48 hours of completing the last chemotherapy cycle, before any supportive psychological nursing approach was initiated. Post-care refers to the follow-up assessment conducted 4 weeks (± 3 days) after discharge, which corresponds to the completion of the 4-week telephone follow-up period. All participants were evaluated at these two fixed time points. ^*^p < 0.05 compared to pre-care within the same group (paired t-test). Cohen’s d represents the effect size for the between-group difference at post-care.

### Comparison of Psychological Distress Levels Between the Two Groups of Patients

At the post-discharge follow-up stage (4 weeks after discharge), the DT score was lower in the supportive care group than in the routine care group (*p* < 0.05). Intra-group comparisons showed that the psychological distress level of patients in the supportive care group experienced significantly lower levels of psychological distress at the post-discharge follow-up stage than at the in-hospital baseline stage (within 48 hours after completing chemotherapy) (*p* < 0.05). In contrast, no statistically significant difference was observed between the two time points in the routine care group (*p* > 0.05) (Table 3).

**Table 3.** **Comparison of psychological distress levels before and after nursing care between the two groups (mean ± SD, points)**.

Group	n	Pre-care	Post-care
Supportive care group	76	4.72 ± 1.49	3.18 ± 1.21^*^
Routine care group	76	4.68 ± 1.46	4.56 ± 1.47
t		0.167	6.319
*p*		0.868	<0.001
Cohen’s d			1.03

^*^p < 0.05 compared to pre-care within the same group (paired t-test).

### Comparison of the Degree of Sleep Disorder Between the Two Groups of Patients

At the post-discharge follow-up stage (4 weeks after discharge), the ISI score in the supportive care group was significantly lower than that in the routine care group, with a statistically significant difference (*p* < 0.05). Intra-group comparisons showed that the ISI scores in the supportive care group were significantly lower at the post-discharge follow-up stage than at the in-hospital baseline stage (*p* < 0.05). In contrast, no statistically significant difference was observed between the two time points in the routine care group (*p* > 0.05) (Table 4).

**Table 4.** **Comparison of the degree of sleep disturbance before and after nursing care in the two groups of patients (mean ± SD, points)**.

Group	n	Pre-care	Post-care
Supportive care group	76	10.36 ± 3.04	7.42 ± 2.63^*^
Routine care group	76	10.18 ± 3.01	10.05 ± 3.01
t		0.367	5.736
*p*		0.714	<0.001
Cohen’s d			0.94

^*^p < 0.05 compared to pre-care within the same group (paired t-test).

### Comparison of Disease Coping Strategies Between the Two Groups of Patients

At the in-hospital baseline stage (within 48 hours of completing chemotherapy), there were no statistically significant differences in the two groups’ scores on the three dimensions of confrontation, avoidance, and resignation (*p* > 0.05). At the post-discharge follow-up stage (4 weeks after discharge), the supportive care group scored higher than the routine care group on the confrontation dimension, while scoring lower on the avoidance and resignation dimensions (*p* < 0.05). Intra-group comparisons showed significant improvement in the supportive care group showed at the post-discharge follow-up stage compared to the in-hospital baseline stage (*p* < 0.05). In contrast, no statistically significant difference was observed in the routine care group before and after the nursing care (*p* > 0.05) (Table 5).

**Table 5.** **Comparison of disease coping strategies before and after nursing care in the two groups (mean ± SD, points)**.

Group	n	Confrontation		Avoidance		Resignation	
		Pre-care	Post-care	Pre-care	Post-care	Pre-care	Post-care
Supportive care group	76	16.61 ± 3.28	19.63 ± 3.12^*^	16.88 ± 2.71	14.27 ± 2.58^*^	15.29 ± 2.63	12.84 ± 2.41^*^
Routine care group	76	16.48 ± 3.39	17.97 ± 3.22	16.92 ± 2.74	15.87 ± 2.66	15.31 ± 2.72	13.98 ± 2.74
t		0.240	9.061	0.090	3.746	0.046	2.724
*p*		0.811	<0.001	0.928	<0.001	0.963	0.007
Cohen’s d			0.51		0.61		0.44

^*^p < 0.05 compared to pre-care within the same group (paired t-test).

## Discussion

Although patients with breast cancer enter a relatively stable treatment phase after completing chemotherapy, their psychological burden does not disappear at the same time as the end of treatment [19]. The physical discomfort, worries about disease recurrence, and role changes experienced during chemotherapy may persist after treatment and manifest as depression, anxiety, and so on [20]. Compared with the acute treatment phase, patients are more likely to experience hidden and delayed emotional problems in the post-chemotherapy phase. Without continuous nursing care, emotional problems can be difficult to identify and treat in a timely manner [21].

This study examined patients in the supportive care group and the routine care group at the post-discharge follow-up stage (4 weeks after discharge). Patients in the supportive care group had lower PHQ-9 and GAD-7 scores than those in the routine care group. No significant differences were found at the in-hospital baseline stage. This improvement in emotional state may be attributed to nurses’ active identification of emotional problems and their provision of continuous communication during daily care. Through multiple one-to-one emotional communication, listening to the patient’s concerns and encouraging them to express their true feelings, patients can obtain emotional support during the nursing process [22]. Compared with the routine inquiry method used by nurses in the routine care group, nurses in the supportive care group pay more attention to the communication process itself. By asking open-ended questions and repeatedly confirming the patient’s emotional state, patients can feel understood and valued. This continuous emotional communication helps patients to gradually release psychological pressure during the nursing process, thereby improving depression and anxiety symptoms [22]. In addition, the nurses cited previous patients’ rehabilitation cases in the communication, helping the patient to realise that their emotional reaction was universal and alleviating their loneliness and feelings of helplessness. This is also an important basis for nursing practice in relation to the decline in emotional scores in the supportive care group [21]. Meanwhile, the results also showed that patients in the supportive care group experienced lower levels of psychological distress than those in the routine care group. This is because, in nursing practice, nurses follow up with patients through multiple channels, communicating continuously and paying attention to their living conditions. This ensures that patients can maintain stable contact with nurses during the post-chemotherapy stage. This continuous nursing presence reduces patients’ anxiety about the unknown and their overall psychological tension [23]. In contrast, the nursing content of the routine care group mainly focuses on treatment-related matters, with relatively limited attention given to patients’ psychological and living situations during follow-up. When experiencing discomfort or psychological distress, patients lack stable communication channels, which may lead to an insignificant decrease in psychological distress levels [24].

Sleep problems are a common but easily overlooked nursing problem for patients with breast cancer after chemotherapy [25, 26]. In this study, sleep disorder in the supportive care group was significantly reduced. This was due to the nursing staff conducting a detailed analysis of the patients’ sleep problems by asking specific questions about sleep duration, night-time awakenings and pre-sleep behaviour during the nursing process [27]. However, the exact mechanisms linking supportive psychological nursing to sleep improvement remain speculative. Potential pathways include reduced anxiety-related hyperarousal and improved sleep hygiene behaviours; however, these were not directly measured in this study. Based on this, the nursing staff guided the patients to establish a fixed routine, reduce pre-sleep stimulating behaviours and continuously monitor improvement. This nursing method, which focused on behaviour adjustment, enabled patients to gradually develop regular sleep habits, alleviating their sleep disorders. In contrast, the routine care group lacked systematic nursing guidance for sleep problems and experienced limited improvement in sleep status [28].

The results also showed that patients in the supportive care group scored higher on the coping dimension and lower on the avoidance and yielding dimensions. This change was closely related to nurses’ guidance methods in emotional communication and health education. By repeatedly emphasising the active role of patients in the recovery process, nurses encouraged patients to focus on modifying their own behaviours rather than passively enduring the impact of the disease. This enabled patients to gradually develop a more positive coping attitude [29]. Furthermore, when communicating with patients and their families, nurses avoided using negative or threatening language. Instead, they provided factual explanations and life guidance to help patients develop a rational understanding of the disease process. This reduced the occurrence of avoidance and negative coping behaviours [30, 31]. It should be noted that the decline in PHQ-9, GAD-7 and ISI scores in this study was generally mild, suggesting that supportive psychological nursing has limited short-term effectiveness in improving symptoms. Considering that the treatment duration in this study was relatively short, and that the subjects’ baseline levels of psychological symptoms were mainly mild, the treatment effect was reflected more in stabilising and mildly improving symptoms than in substantially reversing them. These results suggest that supportive psychological care plays a role in buffering and delaying symptom progression in the post-chemotherapy stage. However, its long-term clinical value still needs to be verified in studies with longer follow-up periods and more intensive treatment.

However, due to the observational design and lack of confounding control, it is not possible to draw causal conclusions. These observations needed to be validated by prospective, randomised controlled trials with longer follow-up periods and standardised nursing approaches.

Limitations: Several limitations should be acknowledged. Firstly, this was a retrospective study based on existing nursing records. Therefore, any observed differences should be interpreted as associations rather than as the result of the nursing approach itself. Grouping based on documentation completeness may reflect baseline differences (e.g., motivation or family support) rather than the nursing strategy itself. Furthermore, the lack of quality control in the extraction of data from routine records and variations in documentation standards may have introduced measurement bias. Secondly, while the hospital information system contained detailed records of potential confounders such as chemotherapy regimens, hormonal therapy, targeted therapy, pain intensity and social support, these variables could not be adjusted for due to the absence of multivariable analysis. Thus, the observed associations may be attributable to unmeasured confounders. Thirdly, the follow-up period was short (4 weeks post-discharge). Longer-term psychological outcomes remain unknown, and the durability of improvements cannot be assessed. Fourthly, this was a single-centre study conducted in one oncology department, which limits its generalisability to other settings or populations. Fifthly, the sample size was modest (76 per group). Although a priori power calculation suggested that the sample size was adequate for detecting moderate effects, the retrospective design may introduce residual confounding factors, and the modest sample size precludes robust subgroup analyses. Sixthly, as data were extracted from routine nursing records without prospective standardisation, measurement bias may have occurred, particularly with regard to the recording of supportive psychological care components. Therefore, the findings should be interpreted as exploratory and hypothesis-generating rather than confirmatory.

## Conclusions

This retrospective study found that supportive psychological nursing care was associated with lower depression and anxiety scores, reduced psychological distress, improved sleep quality and more positive coping strategies in patients with breast cancer after chemotherapy. These results imply a potential link between symptom-targeted supportive psychological nursing care and better psychosocial outcomes.

## Availability of Data and Materials

The datasets used and/or analyzed during the current study are available from the corresponding author on reasonable request.

## References

[1] Amato O, Guarneri V, Girardi F (2023). Epidemiology trends and progress in breast cancer survival: earlier diagnosis, new therapeutics. *Current Opinion in Oncology*.

[2] Ko EB, Jang JB, Hwang DS (2025). Developing of an Integrative Medicine Inpatient Care Program for Breast Cancer Patients Post-Chemotherapy. *Perspectives on Integrative Medicine*.

[3] Javan Biparva A, Raoofi S, Rafiei S, Masoumi M, Doustmehraban M, Bagheribayati F (2023). Global depression in breast cancer patients: Systematic review and meta-analysis. *PLoS ONE*.

[4] Tao L, Xiang Y, Zeng X, Fu L, Li J, Chen H (2024). Psychological-Distress Factors in Patients With Breast Cancer: A Qualitative Meta-Synthesis. *Journal of Clinical Nursing*.

[5] Tao L, Xiang Y, Zeng X, Fu L, Li J, Chen H (2024). Incidence and risk factors for psychological distress in adult female patients with breast cancer: a systematic review and meta-analysis. *Frontiers in Psychiatry*.

[6] Tang WZ, Mangantig E, Iskandar YHP, Cheng SL, Yusuf A, Jia K (2024). Prevalence and associated factors of psychological distress among patients with breast cancer: a systematic review and meta-analysis. *BMJ Open*.

[7] Hewitt M, Herdman R, Holland J, Institute of Medicine (US) and National Research Council (US) National Cancer Policy Board (2004). Meeting psychosocial needs of women with breast cancer.

[8] Hollaender H, Ortner P, Koenig A, Erickson N, Hermelink K, Degenhardt T (2024). Empowering of Oncology Patients and Informal Caregivers: Analysis of an Interdisciplinary Seminar Model for Breast Cancer and Gyneco-Oncological Patients. *Oncology Research and Treatment*.

[9] Dai Q, Liu X, Xu X, Fu Y, She Z, Huang Y (2024). Development of a supportive care framework for breast cancer survivor’s unmet needs: A modified Delphi study. *Journal of Clinical Nursing*.

[10] Wang X, Chen L, Xu Z, Zhu C, Tang Y, Sun J (2025). Effects of a multi-component positive psychological intervention on negative emotions, fatigue and quality of life in patients with breast cancer during initial chemotherapy: a randomized controlled trial. *European Journal of Oncology Nursing: The Official Journal of European Oncology Nursing Society*.

[11] Civilotti C, Lucchini D, Fogazzi G, Palmieri F, Benenati A, Buffoli A (2023). The role of integrated psychological support in breast cancer patients: a randomized monocentric prospective study evaluating the Fil-Rouge Integrated Psycho-Oncological Support (FRIPOS) program. *Supportive Care in Cancer: Official Journal of the Multinational Association of Supportive Care in Cancer*.

[12] Maloney-Newton S, Hickey M, Brant JM, Elsevier (2024). Mosby’s Oncology Nursing Advisor: A Comprehensive Guide to Clinical Practice. 3rd edn. Elsevier. https://hkmu.primo.exlibrisgroup.com/discovery/fulldisplay/alma998430538808061/852HKMU_INST.

[13] van Klinken M, Hafkamp E, Gualtherie van Weezel A, Hales S, Lanceley A, Rodin G (2023). Teaching oncology nurses a psychosocial intervention for advanced cancer: a mixed-methods feasibility study. *Seminars in Oncology Nursing*.

[14] Wang Y, Jiang T (2018). Study on the consistency of Patient Health Questionnaire-9 and Hamilton Depression Scale applied in clinical nursing. *Journal of Qiqihar Medical University*.

[15] Zhou Y, Bi Y, Lao L, Jiang S (2018). Application of GAD-7 in population screening for generalized anxiety disorder. *Chinese Journal of General Practitioners*.

[16] Zhang Y, Zhang H, Song L, Tang L (2010). Application of the Distress Thermometer in screening psychological distress among cancer patients in China. *Chinese Mental Health Journal*.

[17] Bastien CH, Vallières A, Morin CM (2001). Validation of the Insomnia Severity Index as an outcome measure for insomnia research. *Sleep Medicine*.

[18] Shen X, Jiang Q (2000). Report on application of Chinese version of MCMQ in 701 patients. *Chinese Journal of Behavioral Medical Science*.

[19] Yang Y, Zhao Y, Yao K, Li W, Sun H (2025). The influence of cancer treatments on long-term psychological outcomes and quality of life in breast cancer patients. *BMC Cancer*.

[20] Kay DS, Grant DS, Lacey AJ, Kumar DS, Kerin-Ayres MK, Stehn DJ (2024). PREHABILITATION AND SUPPORTIVE CARE IN ONCOLOGY TREATMENT OF BREAST CANCER: PROACTIVE-B. *Journal of Clinical Exercise Physiology*.

[21] Breidenbach C, Heidkamp P, Hiltrop K, Pfaff H, Enders A, Ernstmann N (2022). Prevalence and determinants of anxiety and depression in long-term breast cancer survivors. *BMC Psychiatry*.

[22] Kreitzer N, Fink S, Adeoye O, Kurowski G B, Wade S, Sucharew H (2024). Caregiver Wellness after Traumatic Brain Injury (CG-Well): Protocol for a randomized clinical trial. *Contemporary Clinical Trials Communications*.

[23] Bower JE, Partridge AH, Wolff AC, Thorner ED, Irwin MR, Joffe H (2021). Targeting Depressive Symptoms in Younger Breast Cancer Survivors: The Pathways to Wellness Randomized Controlled Trial of Mindfulness Meditation and Survivorship Education. *Journal of Clinical Oncology: Official Journal of the American Society of Clinical Oncology*.

[24] Rezaee R, Shokrpour N, Rahimi M, Mani A (2020). The effct of peer education on the self-efficy and mental adjustment of breast cancer patients undergoing chemotherapy. *Bangladesh Journal of Medical Science*.

[25] McMahon J, Lautner S, Spadine M, Lee T, Okamoto J (2024). Effectiveness of Susan G. Komen’s national telehealth patient navigation model in reaching underserved populations and reducing cancer-related distress. *Cancer Research*.

[26] Cheng WH, Teo RH, Cheng LJ, Lau Y, Lau ST (2023). Global prevalence of sleep disturbances among breast cancer survivors: a systematic review with meta-analysis. *Sleep Health*.

[27] Dong Y, Wang M, Li W, Zhao K, Cui X, Yang Y (2024). Effect of dexmedetomidine infusion on postoperative sleep disturbances in women with breast cancer: A monocentric randomized-controlled double-blind trial. *Anaesthesia, Critical Care & Pain Medicine*.

[28] Alanazi N, Gu F, Li CS, Lorenz RA, Hong CC (2024). Sleep Quality and Associated Factors Among Survivors of Breast Cancer: From Diagnosis to One Year Postdiagnosis. *Oncology Nursing Forum*.

[29] Tawfik E, Ghallab E, Moustafa A (2023). A nurse versus a chatbot-the effect of an empowerment program on chemotherapy-related side effects and the self-care behaviors of women living with breast cancer: a randomized controlled trial. *BMC Nursing*.

[30] Wei TT, Tian X, Zhang FY, Qiang WM, Bai AL (2020). Music interventions for chemotherapy-induced nausea and vomiting: a systematic review and meta-analysis. *Supportive Care in Cancer: Official Journal of the Multinational Association of Supportive Care in Cancer*.

[31] Bidstrup PE, Johansen C, Kroman N, Belmonte F, Duriaud H, Dalton SO (2023). Effect of a Nurse Navigation Intervention on Mental Symptoms in Patients With Psychological Vulnerability and Breast Cancer: The REBECCA Randomized Clinical Trial. *JAMA Network Open*.

